# Reprogramming human adipose tissue stem cells using epidermal keratinocyte extracts

**DOI:** 10.3892/mmr.2014.2711

**Published:** 2014-10-21

**Authors:** FENG XIE, XINJIE TANG, QUN ZHANG, CHENLIANG DENG

**Affiliations:** 1 Department of Plastic Surgery, Shanghai Ninth People’s Hospital, Shanghai Jiaotong University, Shanghai 200011, P.R. China; 2 Department of Plastic and Reconstructive Surgery, Huashan Hospital, Fudan University School of Medicine, Shanghai 200040, P.R. China; 3 Department of Plastic Surgery, Shanghai Sixth People’s Hospital, Shanghai Jiaotong University, Shanghai 200233, P.R. China

**Keywords:** reprogramming, adipose tissue stem cell, epidermal keratinocytes, cell extract

## Abstract

Human adipose tissue stem cells (ATSCs) can differentiate into various types of cell in response to lineage-specific induction factors. Reprogramming cells using nuclear and cytoplasmic extracts derived from another type of somatic cell is an effective method of producing specific types of differentiated cell. In the present study, the ability of reprogrammed ATSCs to acquire epidermal keratinocyte properties following transient exposure to epidermal keratinocyte extracts was demonstrated. Reversibly permeabilized ATSCs were incubated for 1 h in nuclear and cytoplasmic extracts from epidermal keratinocytes, resealed with CaCl_2_ and cultured. ATSC reprogramming is demonstrated by nuclear uptake of epidermal keratinocyte extracts. After one week of exposure to extracts, ATSCs underwent changes in cell morphology, cell-specific genes were activated, and epidermal keratinocyte markers including K19 and K1/K10 (markers of stem cells and terminally differentiated keratinocytes, respectively) were expressed. This study indicates that the reprogramming of ATSCs using nuclear and cytoplasmic extracts from epidermal keratinocytes is a viable option for the production of specific types of cell.

## Introduction

Regarding treatments for the full spectrum of skin defects, autologous skin grafts are the best option. One major limiting factor of autologous skin grafts is the availability of autologous skin. In recent years, there have been significant advancements in tissue-engineered skin replacements (1–3); however, a good source of autogeneic cells remains to be determined. Nuclear reprogramming offers a promising method, whereby extracts from a specific type of cell are introduced into donor cells and the donor cells subsequently acquire the characteristics of the cells from which the extracts were derived (4). This approach consists of incubating reversibly permeabilized donor cells in nuclear and cytoplasmic extracts derived from another type of somatic cell. Following exposure to the extracts, the donor cells are resealed and subsequently cultured. The extracts are considered to provide the regulatory components required to initiate a transcriptional program specific to the target cell type. Through reprogramming, the epigenetic state of somatic cells can be changed to mirror the epigenetic status of the cell types essential for the treatment of clinical diseases. This approach has been demonstrated for a number of specific types of cell. Håkeliien *et al* (5) reported that 293T cells permeabilized with the cholesterol-binding toxin Streptolysin O (SLO) and exposed to Jurkat T cell extracts were induced to adopt T cell-specific properties. These T cell-specific properties were maintained for ~100 population doublings *in vitro*, suggesting that the Jurkat extracts induced nuclear reprogramming to an extent. Similarly, extracts derived from rat cardiomyocytes have been shown to elicit expression of cardiomyocytic markers in human adipose stem cells (6). These results indicate that nuclear and cytoplasmic extracts from one target cell type can induce some nuclear reprogramming in other types of cells.

In previous reprogramming studies, the type of donor cell used is often mesenchymal stem cells (MSCs) from adult tissues or fibroblasts, which are considered to be able to differentiate outside their predicted developmental lineage (7,8). Human adipose tissue obtained by liposuction has previously been identified as a convenient alternative source for MSCs. Adipose tissue has been processed to obtain a fibroblast-like population of cells or adipose tissue stem cells (ATSCs), which are easily isolated by differential sedimentation from processed liposuction material as previously reported by Zuk *et al* (9). ATSCs and bone marrow-derived mesenchymal stem cells have the capacity for renewal and can be cultured for several months *in vitro* with a regular doubling time and low level of senescence. ATSCs are able to differentiate into multiple cell lineages including adipocytes, osteoblasts, chondrocytes, neurons, and multinucleated myocytes in response to lineage-specific induction factors (9–11). ATSCs migrate, engraft, and differentiate into functional cells *in vivo*.

In the present study, the possibility of using epidermal keratinocyte cell extracts to promote differentiation of ATSCs was investigated.

## Materials and methods

### Isolation and culture of human ATSCs

ATSCs were isolated from liposuction aspirates from subcutaneous adipose tissue sites obtained from male and female subjects undergoing selective procedures in the Departments of Plastic Surgery at Shanghai Ninth People’s Hospital, Shanghai Third People’s Hospital and Shanghai Sixth People’s Hospital, Shanghai Jiaotong University, Shanghai China). For all patients, written informed consent was obtained from the patient or patient’s family and the study was approved by the the Ethical Committee of Shanghai Sixth People’s Hospital. The lipoaspirate was washed 3–4 times with phosphate-buffered saline (PBS) to remove red blood cells and tissue debris. Washed adipose tissue was suspended in an equal volume of PBS supplemented with 1% bovine serum (Hyclone, Logan, UT, USA) and 0.075% collagenase type I (Worthington, Lakewood, NJ, USA), prewarmed to 37°C and then continuously agitated on a shaker at 37°C for 1 h. Collagenase activity was neutralized by adding an equal volume of Dulbecco’s modified Eagle’s medium (DMEM; Gibco-BRL, Carlsbad, CA, USA) with 10% fetal bovine serum. The tissue was centrifuged for 5 min at 197 × g at room temperature. The supernatant, which contained the mature adipocytes, was aspirated. Following centrifugation, cell pellets were resuspended in culture medium, added to tissue culture dishes, and cultured for 24 h at 37°C in 5% CO_2_. Unattached cells and debris were then removed, and fresh medium (DMEM/Ham’s F-12; Gibco-BRL) containing 10% fetal bovine serum (Hyclone) was added to the adherent cells. This initial passage of the primary cell culture was referred to as passage 0. These cells were cultured to 75–90% confluency, passaged using a 0.25% trypsin-ethylenediaminetetracetic acid (EDTA) solution (Sigma-Aldrich, St. Louis, MO, USA) and plated at a density of 5,000 cells/cm^2^ (passage 1). Cell viability and the number of cells at the time of passage were determined using trypan blue (Sigma-Aldrich) exclusion and hemacytometer cell counts respectively. The culture medium was replaced twice a week. Following passage 4, cells were passaged repeatedly once 75–90% confluency was achieved.

### Human epidermal keratinocyte extracts

Human epidermal keratinocytes were obtained from normal skin biopsied from the foreskin or other sites. The skin was washed 3–4 times with PBS, the majority of the subcutaneous tissue was removed with surgical scissors, and the remaining skin was minced finely into pieces <1 mm in diameter. The skin fragments were digested with 2.4 U/ml Dispase (Roche, Mannheim, Germany) at 37°C with occasional agitation. Following 2 h of digestion, the dermis was removed from the epidermis. The epidermal keratinocytes were dissociated from the epidermis via incubation with 0.05% trypsin for 15 min and the cells were cultured in keratinocyte serum-free medium (KSFM; Gibco-BRL). The epidermal keratinocytes were frozen in liquid nitrogen and stored at −80°C for no longer than four weeks. To prepare the epidermal keratinocyte extracts, cells were thawed on ice and cold lysis buffer (50 mmol/l NaCl, 5 mmol/l MgCl_2_, 20 mmol/l 4-(2-hydroxyethyl)-1-piperazineethanesulphonic acid, pH 8.2, and 1 mmol/l dithiothreitol) was added to the cells. Cells were pelleted at 87 × g and resuspended in 1.5 volumes of cell lysis buffer (Beyotime, Jiangsu, China) containing protease inhibitors (Beyotime). Cells were homogenized by pulse-sonication until the cells and their nuclei were completely lysed (monitored by phase contrast microscopy; Nikon TS100, Tokyo, Japan) and the lysate was sedimented at 21,890 × g for 15 min at 4°C. The supernatant was collected and either used fresh or snap-frozen in liquid nitrogen and stored at −80°C. The pH was measured with the aid of a micro-pH meter (Sentron SI600, Roden, The Netherlands) and protein concentration of the extract was measured using The microplate BCA method using a BCA protein assay kit (Pierce, Rockford, IL) according to the manufacturer’s instructions.

### Cell permeabilization

ATSCs were washed in cold PBS and in cold Ca^2+^- and Mg^2+^-free Hanks’ balanced salt solution (HBSS; Gibco-BRL). The cells were resuspended in aliquots of 20,000 cells per 15.5 μl HBSS, and 4.5 μl SLO (Sigma; 100 g/ml stock diluted 1:100 in ice-cold HBSS) was added to yield a final SLO concentration of 230 ng/ml. Samples were incubated for 50 min at 37°C, and then sedimented at 22 × g for 15 min at 4°C. The cells were resuspended in culture medium for use. In each experiment, permeabilization efficiency was evaluated by monitoring uptake of a fluorescein isothiocyanate (FITC)-protein (AnaTag™ 5-FITC Protein Labeling Kit ^*^Ultra Convenient^*^; AnaSpec Inc., Freemont, CA, USA) in a separate sample. The protein was a mixer that included all human epidermal keratinocyte extract proteins. To reseal plasma membranes, cells were plated for 2 h in RPMI-1640 medium containing 10% fetal calf serum and 2 mM CaCl_2_. Cells that failed to reattach were removed during medium replacement. Permeabilization of live cells was examined by fluorescence microscopy (Olympus BX51, Tokyo, Japan).

### Incubation of ATSCs in epidermal keratinocyte extracts

The supernatant was removed when the ATSCs wre cultured in passage 6, and ATSCs were suspended in 20 μl epidermal keratinocyte extract containing an ATP regenerating system (Sigma-Aldrich) and 1 mM of each NTP (ATP, CTP, GTP and UTP; Sigma-Aldrich). The cells were incubated for 1 h at 37°C in a water bath with occasional agitation, allowing for the keratinocyte extract to permeate the ATSCs. To reseal plasma membranes, the extract was diluted in DMEM medium containing 10% FCS, antibiotics (Gibco-BRL), and 2 mmol/l CaCl_2_. Following centrifugation at 197 × g for 5 min, the cells were transferred to culture dishes, and fresh KSFM medium was added. Dead cells were removed through decanting, and the remaining cells were cultured until use. Control cells were permeabilized through exposure to NaCl rather than epidermal keratinocyte extracts.

### Cell viability analysis

The viability of cultured or reprogrammed ATSCs was measured using the Cell Counting kit-8 (Dojindo, Kumamoto, Japan) according to the manufacturer’s instructions. Each experiment was performed in triplicate.

### Laser scanning confocal microscopy

Cultured ATSCs were seeded on a glass slide and incubated with FITC-labeled epidermal keratinocyte protein. At different time points, the cells were fixed with acetone for 10 min at room temperature. Following fixation, the cells were observed using scanning confocal microscopy (TCS-SPE, Leica Microsystems GmbH, Wetzlar, Germany).

### Immunofluorescence

The cells were washed in PBS and fixed with 95% acetone for 15 min. Following fixation, the cells were washed in PBS, permeabilized with 0.25% Triton X-100 and 5% dimethylsufoxide (DMSO)-PBS for 10 min at room temperature and washed in PBS. Subsequently, 0.75/1.5% PBS-H_2_O_2_ was added to the cells for 15 min at 37°C, the cells were washed in PBS, and blocked in 10% sheep serum albumin (Abcam, Cambridge, UK). The cells were incubated overnight with a 1:200 dilution of the primary monoclonal antibodies (mAbs; mouse IgG, K19, involucrin and K1/10 Santa Cruz Biotechnology, Inc, Santa Cruz, CA, USA) and subsequently incubated with the secondary antibody (FITC-conjugated goat anti-mouse antibody; Santa Cruz Biotechnology, Inc.) at 37°C for 30 min. Images were acquired using an Olympus BX51 microscope (Olympus, Tokyo, Japan) and the AnalySIS software, version 1.8 (Soft Imaging Systems, Olympus) and processed with Adobe Photoshop CS5 (Adobe, Mountain View, CA, USA).

### Fluorescence-activated cell sorting (FACS) analysis

Flow cytometric analysis was performed on ATSCs incubated in epidermal keratinocyte extracts and the control cells cultured from passages 0 through 4. Cells were trypsinized and centrifuged for 3 min at 97 × g in an Eppendorf centrifuge. The pellets were resuspended in PBS. The samples were fixed in a solution of 70% ethanol and incubated overnight with 1% Tween-20. The cells were washed in PBS containing 1% bovine serum albumin, and then incubated for 45 min at room temperature with the primary mAbs K19, involucrin and K1/10. Following washing, the secondary antibody (phycoerythrin-conjugated goat anti-mouse antibody, Santa Cruz Biotechnology, Inc.) was added to the cell suspension and incubated for 30 min at room temperature. Cells were washed once more and resuspended in PBS prior to analysis. Cells were analyzed using a FACSCalibur flow cytometer (BD Biosciences, San Jose, CA, USA). Gates were set based on stainings with combinations of relevant and irrelevant mAbs so that no more than 1% of the cells were positive using irrelevant antibodies. Flow cytometric experiments were repeated three times.

### RNA extraction and reverse transcription-polymerase chain reaction (RT-PCR)

Total RNA was extracted from cells using TRIzol (Invitrogen, Gaithersburg, MD, USA), treated with DNase I (Qiagen, Venlo, Netherlands), and processed according to the manufacturer’s instructions. For cDNA synthesis, 1–5 μg total RNA was reverse transcribed (1 h at 37°C) using oligo-dT primers. RT-PCR was performed using the following primers: Forward, 5′-GACGGGAGGGCGAGAAATG-3′, and reverse, 5′-GCCATAGGACATCTGGGAAGC-3′ (246 bp) for K19; forward, 5′-CCAGCCTTCTACACCTCAC-3′, and reverse, 5′-ACCCATTCCTCCCACTCC-3′ (241 bp) for involucrin; forward, 5′-TCCAAGGAAATGGCAACTCA-3′, and reverse, 5′-AGGAACGGCAGGCGAGAT-3′ (288 bp) for K1/10; and forward, 5′-GCGGGAGGGCGAGAAATGA-3′, and reverse, 5′-CGATAGGACATCTGGGAAGCC-3′ (280 bp) for β-actin. PCR conditions were as follows: 94°C for 3 min, 28 cycles of 94°C for 30 sec, 55°C for 30 sec and 72°C for 45 sec, followed by 72°C for 5 min. Each sample (2.5 μl cDNA in a total reaction volume of 25 μl) was run in triplicate. RT-PCR products were analyzed by electrophoresis using a 1% agarose gel. Gels were stained with ethidium bromide and photographed (Image Master VDS, Amersham Pharmacia Biotech, USA) under a UV lamp.

### Statistical analysis

The data are presented as the mean ± standard error of the mean (SEM). One-way analysis of variance test was performed by SAS 6.12 (Software, Inc., San Diego, CA, USA). Mortality was evaluated by the log rank method. P<0.05 was considered to indicate a statistically significant difference.

## Results

### Uptake of FITC-labeling protein by ATSCs

**Figure 1:**
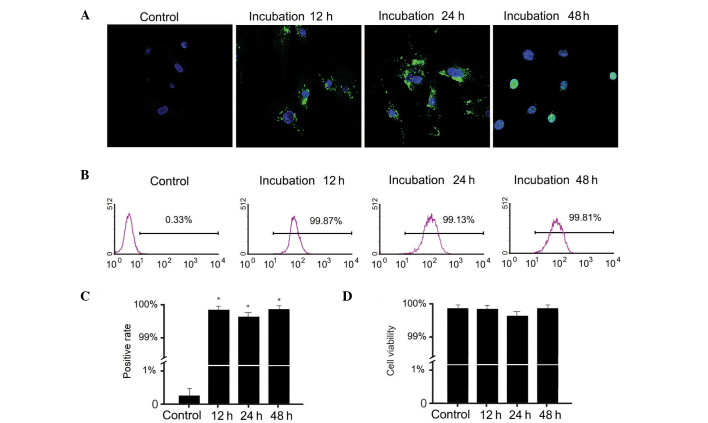
Adipose tissue stem cells (ATSCs) uptake epidermal keratinocyte extracts *in vitro*. (A) At 12 and 24 h post-incubation, the green fluorescence of fluorescein isothiocyanate-labeled cell extracts appeared in the cell cytoplasm. (B) Analysis of cells containing keratinocyte extracts by cell flow cytometry. (C) Statistical analysis of keratinocyte extract uptake rate by ATSCs (^*^P<0.05 vs. the control). (D) Statistical analysis of cell viability after uptake of keratinocyte extracts by ATSCs.

Incubation of ATSCs with epidermal keratinocyte extracts containing FITC-labeling protein resulted in complete cytoplasmic localization of FITC-labeling protein after 12–24 h, followed by nuclear localization after 48 h (Fig. 1A). After 12 h, the SLO-treated and cultured ATSCs displayed bright intracellular FITC fluorescence (Fig. 1A) indicating that a high proportion of cells took up the FITC-labeling protein and resealed. As determined by FACS analysis, the rate of FITC-labeling protein uptake in ATSCs was ~99% (Fig. 1B and C). Cell viability assays demonstrated that uptake of epidermal keratinocyte extracts does not affect cell viability (Fig. 1D).

### Alteration in cell morphology

**Figure 2:**
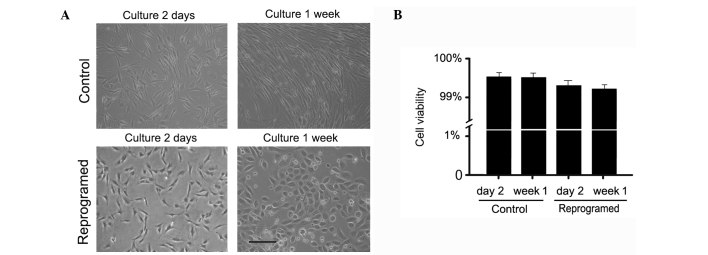
Observations of ATSC morphology and viability in the reprogramming process. (A) Morphology of ATSCs exposed to epidermal keratinocyte extracts after 2 days and 1 week (scale bar, 100 μm). (B) Statistical analysis of cell viability between keratinocyte extracts uptake ATSCs and control cells. adipose tissue stem cell, ATSC.

Permeabilized ATSCs were exposed for 1 h to epidermal keratinocyte extracts, resealed, and expanded in KSFM culture medium. After one day, cultured ATSCs changed shape and acquired a rounder morphology associated with a marked reduction in cell size. After one week of exposure to epidermal keratinocyte extracts, ATSCs tended to resemble epidermal keratinocytes and exhibited different cell sizes that were closely packed (Fig. 2A). This morphology was observed for several weeks. No such changes were observed in the control permeabilized ATSCs. Statistical analysis of cell viability showed no significant difference between the reprogrammed and control cells (Fig. 2B).

### Expression of epidermal keratinocyte markers

**Figure 3:**
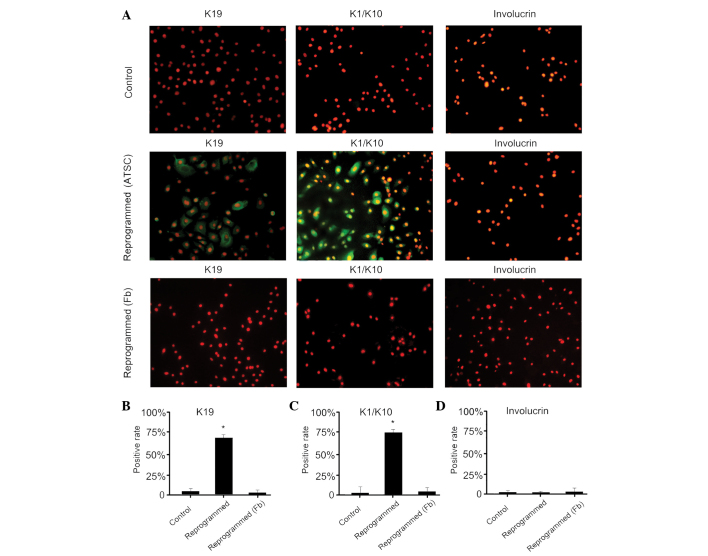
Observations of epidermal keratinocyte protein expressions in reprogrammed (ATSCs) (A) In reprogrammed ATSCs, K19 and K1/10 demonstrate positive expression, while involucrin was not detected by immunofluorescence. In reprogrammed Fbs, K19, K1/10 and involucrin were not detected. (B) Statistical analysis of reprogrammed and control cells expressing K19 (^*^P<0.05 vs. the control). (C) Statistical analysis of reprogrammed and control cells expressing K1/K10 (^*^P<0.05 vs. the control). (D) Statistical analysis of involucrin expression in reprogrammed and control cells demonstrates that there is no significant difference between the two groups. ATSCs, adipose tissue stem cells; Fb, fibroblast.

Permeabilized and epidermal keratinocyte extract-treated ATSCs were cultured for seven days prior to protein expression analysis. Immunofluorescence analysis revealed that reprogrammed cells expressed K19, a marker of epidermal stem cells, and K1/10, a characteristic of epidermal keratinocytes, but did not express involucrin (Fig. 3). The control cells did not express any of the markers analyzed. These results indicate that human ATSCs can be induced to take on properties of epidermal keratinocytes after a transient exposure to nuclear and cytoplasmic extracts from epidermal keratinocytes.

### mRNA expression in reprogrammed ATSCs

**Figure 4:**
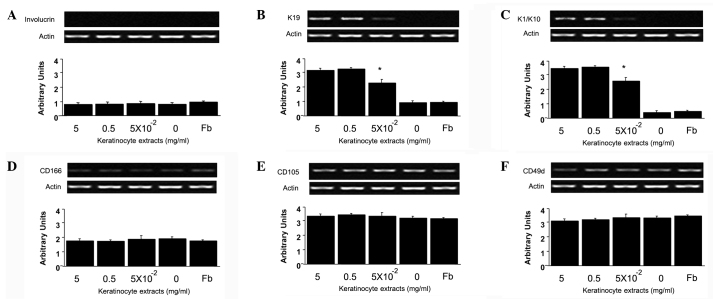
Observations of epidermal keratinocyte mRNA expression in reprogrammed adipose tissue stem cells and fibroblasts (Fb). Representative mRNA expression and statistical analysis of (A) involucrin, (B) K19, (C) K1/K10, (D) CD166, (E) CD105 and (F) CD49d. Fb, fibroblast.

The gene expression levels of K19, K1/10, involucrin, CD166, CD105 and CD49d were analyzed by RT-PCR. The results are shown and summarized in Fig. 4. Involucrin mRNA was not detected in the ATSCs exposed to epidermal keratinocyte extracts (Fig. 4A). As expected, K19 and K1/10 mRNAs were expressed after treatment with ≥5×10^−2^ mg/ml of epidermal keratinocyte extracts, indicating that detection of K19 and K1/10 in ATSCs was dependent on extract protein concentration (Fig. 4B and C). In all cell groups, CD166, CD105 and CD49d mRNA were detected (Fig. 4D–F).

## Discussion

ATSCs can be easily obtained in large quantities from processed liposuction material by differential sedimentation (9,10). The cells that are obtained from lipoaspirates most likely represent a stem cell population. These cells express surface markers which are characteristic of ATSCs and are able to differentiate into bone, cartilage and fat, strongly suggesting they are ATSCs (9). By altering the epigenetic status of somatic cells, it is possible to produce cells that are essential for the treatment of clinical diseases. It has been shown that when nuclear and cytoplasmic extracts from donor cells are introduced into target cells, the target cells can express characteristics of the donor cells (12).

During organ development, different cell types have different epigenetic states that determine the on or off state of specific genes. Changing the epigenetic status of mature cells allows for the production of cells that are essential for the treatment of clinical diseases. Nuclear reprogramming is a method that can change the epigenetic state and function of cells and it is of great medical interest as it has the potential to generate a source of patient-specific cells. The ability to reprogram specific somatic cells into other types of cells has previously been demonstrated (5,6,11). In the present study, reversibly permeabilized ATSCs were incubated for 1 h in nuclear and cytoplasmic extracts from human epidermal keratinocytes, resealed with CaCl_2_ and cultured. Following ~2 h of incubation, the FITC-labeling protein of epidermal keratinocyte extracts began to gradually permeate into the ATSCs. After ~12–24 h, FITC fluorescence was found throughout the cytoplasm and 48 h later the fluorescence was observed in the nucleus. Using FACs analysis, the ATSC uptake rate of FITC-conjugated protein was determined to reach 99%, thus demonstrating the feasibility of this method. These results demonstrate that ATSC reprogramming through exposure to nuclear and cytoplasmic epidermal keratinocyte extracts is a promising method. The results of multiple experiments support the hypothesis that differentiation is induced in ATSCs. Changes in cell shape were observed after one day, and cultured ATSCs acquired a rounder morphology associated with a small reduction in cell size. Following one week of exposure to epidermal keratinocyte extracts, ATSCs tended to resemble epidermal keratinocytes and exhibited the pavestone appearance. Immunofluorescence analysis demonstrates that reprogrammed cells expressed K19, an epidermal stem cell marker, and K1/10, an epidermal keratinocyte marker. The control cells did not express any of the markers analyzed. K19 and K1/10 mRNA levels in reprogrammed ATSCs increased with increasing concentrations of protein from epidermal keratinocyte extracts. These results are consistent with the results from the immunofluorescence and FACS analysis. Reprogramming appears to be elicited by direct uptake of factors from the extract, although how the extract affects ATSCs and the nature of the cell extract molecules responsible for induction of ATSC differentiation remain to be investigated.

Notably, K19 and K1/10 were detected in the experiments, while involucrin was not. One possible explanation is the heterogeneity of the ATSC population within a lipoaspirate, similar to the heterogeneity within bone marrow-derived MSCs (13). Only a fraction of ATSCs may be responsive to the differentiation factors provided by the extract.

In the present study, the nature of the cell extract molecules responsible for the induction of ATSC differentiation in the *in vitro* approach remains largely unknown. Transcription factors, signaling pathways and chromatin remodeling complexes are likely involved in differentiation. Previous studies indicate that chromatin remodeling is facilitated by induction of histone H4 hyperacetylation at the IL2 locus in 293T nuclei incubated in Jurkat extracts, which correlates with activation of the IL2 gene (5,14). Furthermore, induced expression of previously repressed genes has also been observed, indicating that the extract is capable of reprogramming the epigenetic state of the 293T genome. Nevertheless, a role for nucleic acids, including small noncoding nuclear RNAs, in promoting changes in gene expression following the incubation of cells in extracts has not been excluded in light of their function in transcriptional regulation (15,16). Mikkelsen *et al* (17) suggested that certain cells may become trapped in partially reprogrammed states due to incomplete repression of transcription factors and that DNA demethylation is an inefficient step in the transition to pluripotency. They demonstrated that RNA inhibition of transcription factors facilitates reprogramming and that treatment with DNA methyltransferase inhibitors improves the overall efficiency of the reprogramming process. In addition, Huangfu *et al* (18,19) demonstrated that histone deacetylase and DNA methyltransferase inhibitors greatly improve the efficiency of reprogramming mouse embryonic fibroblasts by genetic factors. Biochemical fractionation of the extracts is expected to help elucidate the nature of molecules involved in altering cell fate.

In conclusion, the present study determined that chromatin remodeling and transcription factors are key elements involved in the induction of reprogramming. However, certain factors are yet to be elucidated, including how nuclear reprogramming is induced, the mechanism behind induction and how long the developmental programming persists.
